# Chikungunya virus persists in joint-associated macrophages and promotes chronic disease

**DOI:** 10.21203/rs.3.rs-6917990/v1

**Published:** 2025-06-25

**Authors:** Kristen M. Zarrella, Ryan M. Sheridan, Brian C. Ware, Bennett J. Davenport, Mariana O. L. da Silva, Dariia Vyshenska, Aspen U. Martin, Nick A. May, Daniela Weiskopf, Jay R. Hesselberth, Daniel N. Streblow, Alex L. Greninger, Thomas E. Morrison

**Affiliations:** 1 Department of Immunology & Microbiology, University of Colorado School of Medicine, Aurora, CO, USA; 2 RNA Bioscience Initiative, University of Colorado School of Medicine, Aurora, CO, USA; 3 Instituto de Microbiologia Paulo de Goes, Universidade Federal do Rio de Janeiro, Rio de Janeiro, Brazil; 4 Virology Division, Department of Laboratory Medicine and Pathology, University of Washington Medical Center, Seattle, WA, USA; 5 Department of Biochemistry & Molecular Genetics, University of Colorado School of Medicine, Aurora, CO, USA; 6 Center for Vaccine Innovation, La Jolla Institute for Immunology, La Jolla, CA 92037, USA; 7 Department of Medicine, Division of Infectious Diseases and Global Public Health, University of California San Diego (UCSD), La Jolla, CA 92037, USA; 8 Vaccine and Gene Therapy Institute and Division of Pathobiology and Immunology, Oregon National Primate Research Center, Oregon Health and Science University, Beaverton, OR 97006, USA

## Abstract

Arthritogenic alphaviruses including chikungunya, Mayaro, and Ross River viruses cause long-lasting musculoskeletal pain and inflammation. However, mechanisms driving chronic disease remain poorly understood. Here, we investigated joint-associated tissues in alphavirus-infected mice at a late stage of infection. Utilizing scRNA-seq, spatial transcriptomics, and flow cytometry we identified an accumulation of inflammatory macrophages in joint-associated tissues with elevated *Tnf*, *Nlrp3*, *Il1b,* and *H2-Aa* expression, and these cells harbored CHIKV RNA. Moreover, we identified an accumulation of CD4^+^ T cells in joint-associated tissues, which express *Ifng*. Depletion of CD4^+^ T cells diminished MHC-II expression on joint macrophages, highlighting their potential role in inflammation. In addition, treatment with a small molecule inhibitor of CHIKV replication during chronic disease reduced viral RNA and joint inflammation, suggesting that viral RNA replication promotes chronic joint disease. Our data suggest that macrophages harbor replicating viral RNA and contribute to the sustained joint inflammation associated with chronic alphavirus disease.

Arthritogenic alphaviruses, including chikungunya (CHIKV), Mayaro (MAYV), o’nyong-nyong (ONNV), Ross River (RRV), and Sindbis (SINV) viruses, are globally distributed RNA arboviruses that cause debilitating acute and chronic musculoskeletal disease.^1,2^ Since 2004, CHIKV has re-emerged to cause outbreaks of increasing magnitude and severity in the Indian Ocean region and the Americas.^3–5^ It is estimated that CHIKV causes 35 million annual infections and 2.8 billion people are at risk,^6^ underscoring the ongoing challenge to public health systems. The widespread distribution of CHIKV’s mosquito vectors, *Aedes aegypti* and *Aedes albopictus*,^7^ further emphasizes the risk of continued transmission and future outbreaks.

Acute arthritogenic alphavirus disease onset occurs within 4-8 days of virus exposure, with symptoms including debilitating fever, polyarthralgia, joint swelling, myalgia, headache, nausea, fatigue, and rash.^8,9^ Studies in both humans and animal models, including non-human primates (NHPs) and mice, have shown that arthritogenic alphavirus infection can lead to myositis, tenosynovitis, synovitis, bone erosion, and cartilage damage.^10–16^ Additionally, the acute disease is characterized by myeloid and lymphocytic cellular infiltration in musculoskeletal tissues^17,18^ as well as elevated chemokines that promote immune cell recruitment such as CCL2, CCL4, and CXCL10.^18–21^ Blockade of these chemokines or IL-1β diminished CHIKV-induced bone loss in mice, while CCR2 deficiency caused increased neutrophil infiltration and exacerbated acute disease, suggesting cellular infiltrates and pro-inflammatory factors contribute to joint pathology during acute infection.^13,22,23^

Depending on the outbreak, 10-50% of patients with acute arthritogenic alphavirus disease progress to a chronic form, characterized by persistent or relapsing arthralgia and arthritis that can last for months to years.^24–27^ These chronic symptoms resemble those observed in individuals with rheumatoid arthritis (RA).^28^ Indeed, chronic CHIKV arthritis and RA patients share similar synovial cytokine profiles characterized by elevated levels of IL-1β, IFN-γ, and TNF among others.^29^ Nonetheless, few studies have demonstrated antigen-dependent autoimmune features in patients with chronic CHIKV arthritis like those seen in RA patients, such as anti-nuclear (ANA) or anti-cyclic citrullinated peptide (CCP) antibodies and rheumatoid factor (RF),^30^ suggesting arthritogenic alphavirus disease and RA have distinct immunological mechanisms despite the overlapping disease features.

Persistent CHIKV infection in joint-associated tissues has been hypothesized to contribute to chronic chikungunya disease,^31^ but testing this hypothesis in human patients remains challenging. The joints of the wrists, hands, and feet are the primary sites affected in patients with chronic CHIKV disease,^2,30^ but these sites are difficult to evaluate for the presence of infectious virus, viral RNA, or viral antigen. In rare examples, CHIKV antigen and RNA have been detected in synovial and muscle tissue biopsy specimens collected from patients during the chronic phase of disease.^32,33^ However, other studies failed to detect viral RNA in synovial fluid or tissue biopsies collected from patients diagnosed with chronic chikungunya.^34,35^ More evidence for arthritogenic alphavirus persistence comes from experiments in animal models. In immunocompetent mice, CHIKV, MAYV, ONNV, and RRV RNA, as well as CHIKV antigen, persist in joint-associated tissues for weeks to months following infection,^15,36–44^ with fibroblasts identified as one potential cellular reservoir.^43^ Similarly, in CHIKV-infected macaques, joint, muscle, liver, and lymphoid tissues harbor infectious CHIKV, CHIKV RNA, or CHIKV antigen for weeks after inoculation.^11,14^ Beyond fibroblasts, the cellular sites of viral persistence remain to be fully elucidated, and the role of viral persistence, or the persistence of viral products such as antigen and RNA, in the development of chronic disease is not understood.

To identify cell types that contribute to chronic CHIKV disease, we performed single-cell mRNA sequencing (scRNA-seq) of cells isolated from ankle tissue 4 weeks after CHIKV inoculation, revealing that chronic CHIKV infection in joint tissue transcriptionally resembles other chronic, inflammatory diseases such as RA. This analysis also identified macrophages and fibroblasts as having the greatest transcriptional changes with CHIKV infection, including increased infiltrating macrophages with elevated MHC-II associated genes, *Tnf*, and *Nlrp3* expression. Consistent with prior studies in humans and NHPs,^14,32^ our scRNA-seq analysis identified macrophages as a key reservoir for persistent CHIKV RNA and deep sequencing analysis of viral RNA showed full coverage of the CHIKV genome. Fluorescence activated cell sorting (FACS) of fixed ankle tissue cells confirmed macrophages harbor CHIKV RNA and display elevated expression of *Tnf*, *Il1b*, and *Nlrp3*, and similar results were observed in MAYV- and RRV-infected mice, implicating macrophages as pathogenic effectors of chronic alphavirus joint disease and sites of alphavirus persistence. In addition, our studies identified elevated CD4^+^ T cells that express *Ifng* and perpetuate the activation of macrophages within the joint tissue of CHIKV-infected mice at late times post-infection. Finally, spatial transcriptomics of joint tissue identified both positive-strand and negative-strand CHIKV RNA in fibroblasts and macrophages, suggesting ongoing viral replication during chronic disease. Thus, we utilized a small molecule inhibitor of CHIKV replication during chronic disease, which resulted in reduced viral RNA levels, MHC-II^+^ macrophages, and *Tnf*, *Il1b*, *Nlrp3*, and *H2-Aa* expression, implicating viral RNA replication as a driver of chronic joint inflammation. These studies provide insight into the immunopathologic mechanisms underlying chronic arthritogenic alphavirus disease.

## RESULTS

### Joint-associated tissue displays an inflammatory gene signature during the chronic phase of CHIKV infection

**Figure 1.**
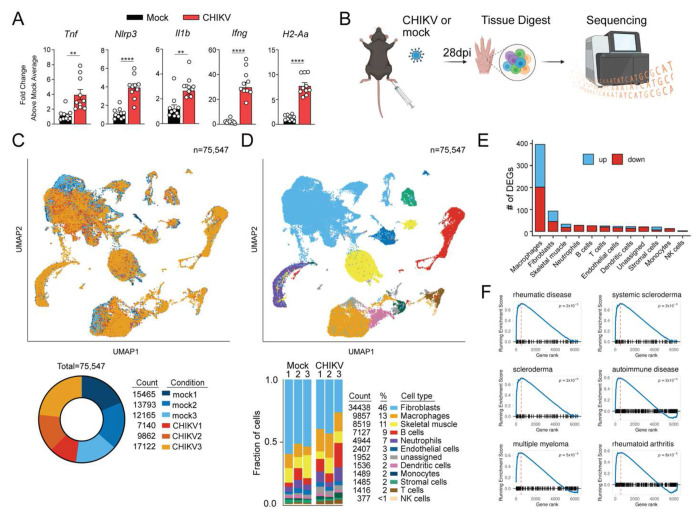
Single cell transcriptomics analysis of joint tissue during the chronic stage of CHIKV infection. (**A**) WT C57BL/6 mice were inoculated with PBS (mock; n=10) or 10^3^ PFU CHIKV (n=10) in the left rear footpad. At 28 dpi, inflammatory gene expression in ankle joints was quantified by RT-qPCR. Data are from 2 independent experiments. *P* values were determined by unpaired Students t test. **, *P*<0.01; ****, *P*<0.0001. (**B**) Schematic of experimental design. WT C57BL/6 mice were inoculated with PBS (mock; n=3) or 10^3^ PFU CHIKV (n=3) in the left rear footpad. At 28 dpi, ankle joint-associated single cells were analyzed by scRNA-seq. (**C**) UMAP and pie chart showing condition identity (CHIKV: red colors, mock: blue colors) and cell counts for 3 replicates. (**D**) UMAP and bar graph showing cell type annotations for each sample. (**E**) Bar graphs showing the number of genes upregulated and downregulated in CHIKV-infected mice compared with mock-infected mice (Blue/top bar: upregulated, red/bottom bar: downregulated). (**F**) GSEA for genes upregulated in macrophages from CHIKV-infected mice.

To elucidate mechanisms of chronic CHIKV disease, we used an established wildtype (WT) C57BL/6 mouse model of CHIKV-induced musculoskeletal tissue inflammation and injury that recapitulates many of the hallmarks of arthritic disease observed in CHIKV-infected patients.^15^ Initially, we evaluated joint-associated tissue for signs of inflammation during the chronic stage (i.e., 28 days post-infection; dpi). This time point was selected based on prior studies demonstrating immune-mediated clearance of an attenuated vaccine strain of CHIKV, but not WT CHIKV strains, from joint-associated tissues by 28 dpi.^41^ Prior studies of acute CHIKV infection in mice showed elevated pro-inflammatory markers such as IL-1β, TNF, NLRP3, MHC-II, and IFN-γ.^18,23,45,46^ Following subcutaneous (s.c.) virus inoculation in the footpad, the ipsilateral ankle of CHIKV-infected mice at the chronic stage of infection also displayed an inflammatory gene expression signature characterized by heightened expression of *Tnf, Nlrp3, Il1b, Ifng,* and *H2-Aa* (MHC-II) (Figure 1A). NLRP3 is a component of the inflammasome involving caspase-1 or caspase-8, which cleave pro-IL-1β to the functional form, IL-1β.^47^ Notably, TNF and IL-1β expression from macrophages have been implicated in cartilage and collagen destruction in rheumatoid arthritis.^48,49^ To evaluate the inflammatory state in joint-associated tissues during the chronic stage with greater resolution, we analyzed cells present in joint-associated tissue using scRNA-seq. WT C57BL/6 mice were inoculated with PBS (mock) or 10^3^ PFU of CHIKV. At 28 dpi, single-cell suspensions were generated from ipsilateral joint-associated tissue and processed for scRNA-seq (Figure 1B). In total, 75,547 cells were recovered from ipsilateral ankle tissue of mock and CHIKV-infected mice (n = 3 mice per group) (Figure 1C). Comparable to previous flow cytometry results,^50,51^ both hematopoietic (35%) and connective tissue cells (62%) comprised the cell populations captured from the ipsilateral ankle (Figure 1D). Macrophages (13%) and B cells (9%) comprised the largest proportions of hematopoietic cells among all cells and were accompanied by neutrophils (7%), dendritic cells (2%), monocytes (2%), T cells (2%) and NK cells (<1%). Macrophages and fibroblasts exhibited the largest number of differentially expressed genes (DEGs) compared with cells from control mice (Figure 1E). Moreover, using gene set enrichment analysis (GSEA) to compare these scRNA-seq data set with publicly available gene lists, we found that genes upregulated in joint-associated macrophages from CHIKV-infected mice showed substantial overlap with rheumatological conditions including RA and scleroderma (Figure 1F).

### Macrophages harbor CHIKV RNA at the chronic stage of infection

**Figure 2.**
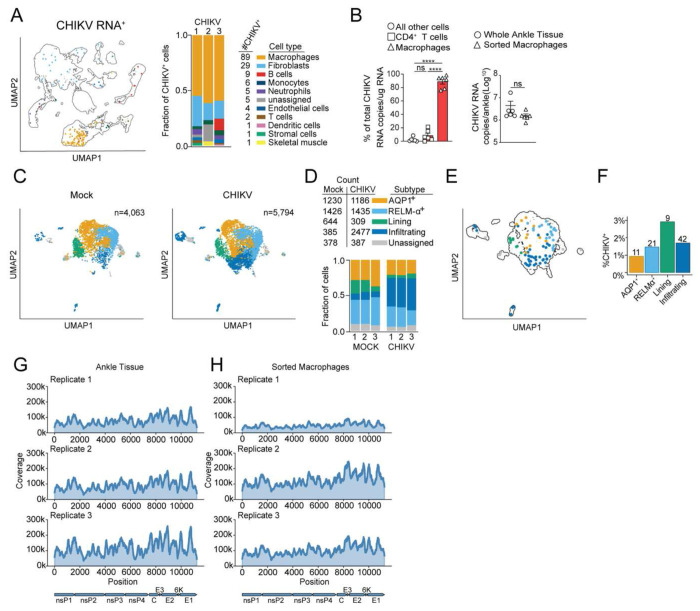
Macrophages harbor CHIKV RNA at the chronic stage of infection. (**A**) UMAP and bar graph showing cells identified as CHIKV RNA^+^. (**B**) WT C57BL/6 mice were inoculated with PBS (mock; n=6) or 10^3^ PFU CHIKV (n=6) in the left rear footpad. At 28 dpi, ankle joint-associated single cells were isolated, stained for flow cytometry (live, singlets, CD45, B220, Ly6G, NK1.1, CD11b, CD11c, CD90.2, and CD4), fixed, sorted, and CHIKV RNA was quantified by RT-qPCR. Graphs show the percent of total CHIKV RNA copies detected within sorted populations (circles: all other cells; squares: T cells; triangles: macrophages) and total CHIKV RNA copies detected in sorted macrophages and whole ankle tissue. Data are from 2 independent experiments. *P* values were determined by one-way ANOVA with Tukey’s multiple comparisons test or unpaired Student’s t test. ****, *P*<0.0001. (**C**) UMAP showing macrophage subsets in mock- and CHIKV-infected mice. (**D**) Bar graph showing the fraction of total macrophages that comprise the different subsets. (**E**) UMAP showing CHIKV RNA^+^ macrophages per subset. (**F**) Frequency of CHIKV RNA^+^ macrophages in each subset. The numbers above each bar indicate the total number of CHIKV RNA^+^ macrophages detected for each subset. (**G-H**) Coverage maps from RNA isolated from (**G**) whole joint-associated tissue or (**H**) sorted joint-associated macrophages from CHIKV-infected mice at 28 dpi.

In prior studies, we and others reported that CHIKV RNA and antigen persist in joint-associated tissues for weeks to months after infection of immunocompetent mice, including in fibroblasts.^15,37,39–41,43,52,53^ In addition, CHIKV antigen has been detected in synovial tissue macrophages from a patient diagnosed with chronic CHIKV disease and in splenic macrophages months after experimental CHIKV infection of NHPs.^14,32^ Several studies found that macrophages can support alphavirus replication, in some cases for weeks, *in vitro*,^18,54–56^ suggesting that macrophages could serve as a viral reservoir. To further define cells that harbor viral RNA during the chronic stage of CHIKV infection in mice, we first evaluated whether our single cell suspensions of ipsilateral ankle tissue captured CHIKV RNA positive cells. We detected similar levels of CHIKV RNA in total RNA extracted from homogenates of intact ipsilateral ankle tissue and single-cell suspensions generated by enzymatic digestion of ipsilateral ankle tissue (Figure S1A). Thus, analysis of CHIKV RNA^+^ cells in our scRNA-seq data should be representative of the CHIKV RNA signal typically detected in joint-associated tissue. Among all cells from joint-associated tissue of CHIKV-infected mice, 152 had at least 1 detected CHIKV RNA copy (0.2% of total cells) for which macrophages (59%) and fibroblasts (19%) expressed the majority (Figure 2A). To confirm that CHIKV RNA is in macrophages, we employed a paraformaldehyde (PFA)-fixed fluorescence activated cell sorting (FACS)-based method to specifically isolate macrophages, CD4^+^ T cells, and all other cells to measure CHIKV RNA and host cell gene expression. At 28 dpi, single cell suspensions were generated from ipsilateral joint tissue, cells were stained with fluorophore-conjugated antibodies, and cells were then fixed with 2% PFA. CD4^+^ T cells, macrophages, and all other cells were sorted as shown in Figure S1B. Total RNA was isolated from sorted cell populations (Figure S1C) and CHIKV RNA was quantified by RT-qPCR. The sorted macrophages contained nearly 90% of the CHIKV RNA signal detected across the three sorted cell populations and the signal detected in the macrophage population was comparable to digested whole ankle tissue (Figure 2B). Collectively, these data suggest that macrophages are the predominant cell types in joint-associated tissue harboring CHIKV RNA during chronic disease.

Prior studies reported that resident macrophages in joint-associated tissues are a diverse population of cell types that function in tissue homeostasis and resolution of inflammation, whereas infiltrating monocyte-derived macrophages promote inflammation and injury.^57,58^ A scRNA-seq study of non-infectious arthritis in mice by Culemann et al.^58^ showed that macrophages in joint-associated tissue could be divided into multiple subsets including CX_3_CR1^+^ lining macrophages, two CX_3_CR1^−^ interstitial macrophage populations (AQP1^+^ and RELM-α^+^), and infiltrating macrophages characterized by expression of *Ccr2* and *Ly6c2*. Among the joint tissue-associated cells we analyzed by scRNA-seq at 28 dpi, we identified similar macrophage subsets in samples from both mock and CHIKV-infected mice (Figures 2C, 2D, and S2A). A similar cell count was evident among lining, unassigned, AQP1^+^, and RELM-α^+^ macrophage subsets in mock and CHIKV-infected mice (Figure 2D). In contrast, the number of infiltrating macrophages was ~6.5-fold higher in joint-associated tissue of CHIKV-infected mice (Figure 2C and 2D). Among the macrophage subsets, the CHIKV RNA^+^ frequency ranged from 1-3% (Figure 2E and 2F).

Next, we performed deep sequencing analysis of the CHIKV RNA detected in joint-associated tissue (Figure 2G) and sorted joint macrophages (Figure 2H). Across all replicates, we detected deep, even coverage across the full-length of the CHIKV RNA genome, suggesting that the complete viral genome is maintained in cells. Analysis of CHIKV alleles that changed by >10% allele frequency between CHIKV sequences recovered from tissues and macrophages with the viral sequence present in the inoculum (Figure S2B) revealed 2 synonymous mutations in one of three replicates from joint-associated tissue (nsP2 T209T 10.2%; nsP4 R521R 10.9%) as well as 2 synonymous (capsid P87P 14.4%; capsid K71K 21.2%) and 1 non-synonymous mutation (6K T47I 10.8%) in one of three replicates from macrophages (Figure S2C), suggesting that adaptive mutations are not essential for persistence of the CHIKV genome in macrophages.

### Macrophages have an inflammatory transcriptional program and elevated levels of MHC-II

**Figure 3.**
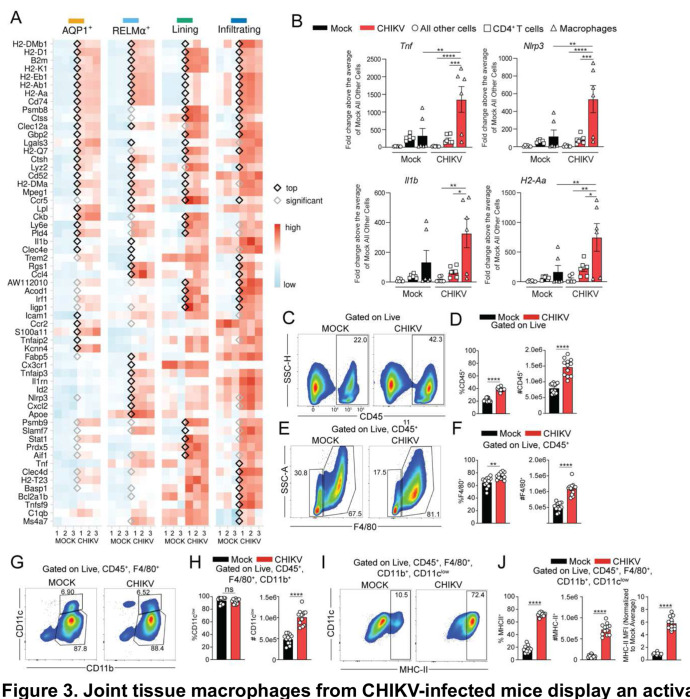
Joint tissue macrophages from CHIKV-infected mice display an activated and pro-inflammatory transcriptional program. (**A**) Heatmap showing genes upregulated in macrophage subsets from CHIKV-infected mice at 28 dpi. Genes with a black diamond are significantly upregulated and are within the top 20 upregulated genes for the subset. Genes with a grey diamond are significantly upregulated in that macrophage subset but are not within the top 20 upregulated genes. (**B**) RT-qPCR analysis of inflammatory gene expression in macrophages, CD4^+^ T cells, and all other cells sorted from ankle joints of mock- (n=6) or CHIKV-infected (n=6) mice at 28 dpi. (**C-J**) WT C57BL/6 mice were inoculated with PBS (mock; n=12) or 10^3^ PFU CHIKV (n=12) in the left rear footpad. At 28 dpi, ankle joint cells were analyzed by flow cytometry. (**C**) Representative flow cytometry plots of CD45^+^ cells among live, singlet cells. (**D**) Frequency and number of CD45^+^ cells. (**E**) Representative flow cytometry plots of F4/80^+^ cells among live, singlet, CD45^+^ cells. (**F**) Frequency and number of F4/80^+^ cells among live, singlets, CD45^+^ cells. (**G**) Representative flow cytometry plots for CD11c^+^ and CD11b^+^ cells among live, singlet, CD45^+^, F4/80^+^ cells. (**H**) Frequency and number of CD11c^low^ cells. (**I**) Representative flow cytometry plots of MHC-II expression on macrophages. (**J**) Frequency and number of MHC-II^+^ macrophages, and MHC-II geometric mean fluorescent intensity (gMFI). Data are representative of 2 independent experiments. *P* values were determined by one-way ANOVA with Tukey’s multiple comparisons test (B) or unpaired Students’ t test (D, F, H, J). *, *P*<0.05; **, *P*<0.01; ***, *P*<0.001; ****, *P*<0.0001.

Analysis of gene expression among all macrophage subsets revealed that those in joint-associated tissue of CHIKV-infected mice displayed elevated expression of numerous genes involved in an inflammatory response including *Ctss, Pld4, Clec4e, Ccl4, Tnfaip2, Tnf, Aip3, Slamf7, Aif1, Cxcl2, Tnf, Il1b,* and *Nlrp3* ^59–62^ (Figure 3A) as well as many genes associated with immune cell infiltration or monocyte migration to sites of inflammation such as *Ccr5*, *Ccr2*, and *Cx3cr1*.^63^ Analysis of gene expression patterns across the different macrophage subsets revealed some differences in the top DEGs (Figure 3A). Macrophages in each subset expressed elevated levels of MHC-II associated genes above their counterparts in control mice such as *H2-Dmb1*, *H2-Eb1*, *H2-Ab1*, *H2-Aa*, and *H2-Dma* and other genes regulated by IFN-γ such as *Psmb8*, *Irf1*, *Icam1*, *Stat1*, *Slamf7*, *Nlrp3*, and *Tnf*.^64–66^ Taken together, these data suggest that macrophages within the joint tissue are comprised of heterogenous subsets that are pro-inflammatory and MHC-II^+^ in CHIKV-infected mice, even at 4 weeks post virus inoculation.

To confirm these findings, macrophages, CD4^+^ T cells, and all other cells were FACS sorted from the joint tissue of control and CHIKV-infected mice at 28 dpi as described above, and gene expression in sorted cells was measured by RT-qPCR. Consistent with our scRNA-seq analysis, macrophages from CHIKV-infected mice displayed increased expression of *Tnf, Nlrp3, Il1b, and H2-Aa* compared to CD4^+^ T cells and all other cells (Figure 3B and Figure S2D). However, only *Tnf*, *Nlrp3*, and *H2-Aa* were elevated above joint macrophages sorted from uninfected control mice. Remarkably, all other cells isolated from the joint tissue of CHIKV-infected mice expressed relatively low levels of these inflammatory genes (Figure 3B), suggesting that macrophages are a primary driver of joint tissue inflammation during the chronic stage of CHIKV infection.

We further evaluated macrophages present in joint-associated tissues at 28 dpi using flow cytometry. There was an increase in CD45^+^ (Figure 3C–D) as well as F4/80^+^ (Figure 3E–F) cells in CHIKV-infected tissue compared with controls. Among live, singlets, CD45^+^, F4/80^+^ cells, we found a heterogenous population of CD11b- and CD11c-expressing cells (Figure 3G). The majority (>80%) were CD11b^+^, CD11c^low^ (Figure 3G–H). We evaluated these cell populations for cell surface expression of MHC-II (Figure 3I–J and Figure S2E–F) and observed the F4/80^+^CD11b^+^CD11c^high^ population expressed similar levels of MHC-II in both mock and CHIKV-infected mice (Figure S2E–F), possibly Langerhans cells (LCs), which constitutively express MHC-II.^67–69^ In contrast, the F4/80^+^CD11b^+^CD11c^low^ cells in CHIKV-infected mice displayed a 4-fold increase in the total number of cells expressing MHC-II, and a 6-fold increase in the surface expression of MHC-II (geometric mean fluorescence intensity; gMFI) compared with the same cells from uninfected mice (Figure 3I–J). Therefore, there is a sustained population of MHC-II^+^ macrophages in joint-associated tissue of CHIKV-infected mice during chronic disease.

### CD4^+^ T cells regulate MHC-II expression on macrophages during chronic CHIKV disease

**Figure 4.**
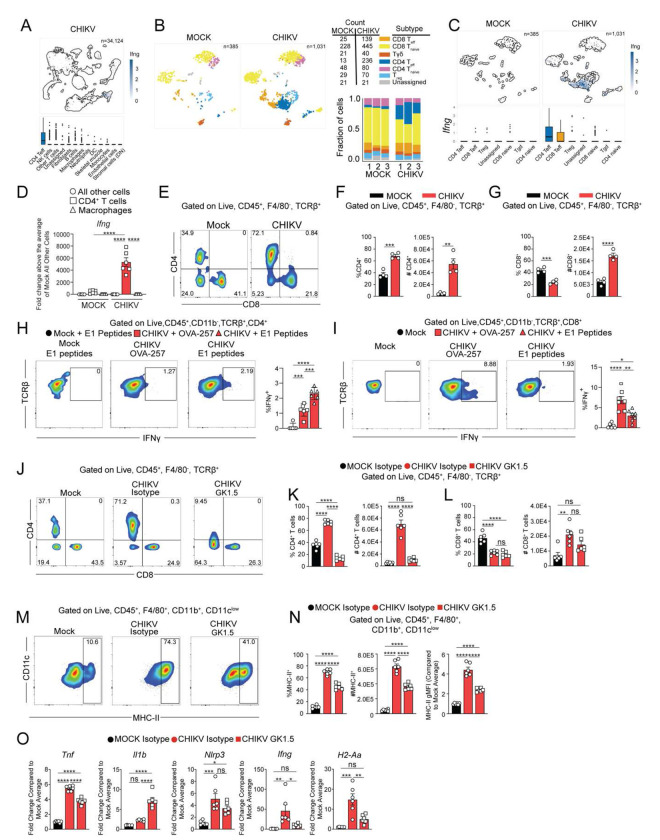
CD4^+^ T cells regulate MHC-II^+^ macrophages during chronic CHIKV joint disease. (**A**) UMAP comparing *Ifng* expression for CD4 T_effs_ cells and all other cell types. (**B**) UMAP showing T cell subsets. Table and bar graph show total counts and fraction for each T cell subset, respectively. (**C**) UMAP showing *Ifng* expression for T cell subsets from mock- and CHIKV-infected mice. (**D**) RT-qPCR analysis of *Ifng* expression in macrophages, CD4^+^ T cells, and all other cells sorted from ankle joints of mock- or CHIKV-infected mice at 28 dpi. (**E-G**) WT C57BL/6 mice were inoculated with PBS (mock; n=4) or 10^3^ PFU CHIKV (n=4) in the left rear footpad. At 28 dpi, ankle joint cells were analyzed by flow cytometry. (**E**) Representative flow cytometry plots of CD4^+^ and CD8^+^ cells among live, singlet, CD45^+^F4/80^−^TCRβ^+^ cells. (**F-G**) Frequency and total number of CD4^+^ and CD8^+^ cells among live, singlet, CD45^+^F4/80^−^TCRβ^+^ cells. (**H-I**) WT C57BL/6 mice were inoculated with PBS (n=6) or 10^3^ PFU CHIKV-OVA (n=6) in the left rear footpad. At 28 dpi, ankle joint single cell suspensions were stimulated *ex vivo* with the indicated peptides for 5 h in the presence of brefeldin A. (**H**) Representative flow cytometry plots of IFNγ^+^ cells among live, singlet, CD45^+^CD11b^−^TCRβ^+^CD4^+^ cells. Bar graph shows the frequency of IFNγ^+^ cells in each group. (**I**) Representative flow cytometry plots of IFNγ^+^ cells among live, singlet, CD45^+^CD11b^−^TCRβ^+^CD8^+^ cells. Bar graph shows the frequency of IFNγ^+^ cells in each group. (**J-O**) WT C57BL/6 mice were inoculated with PBS (n=6) or 10^3^ PFU CHIKV(n=6) in the left rear footpad. At 14 dpi, mice were injected intraperitoneally with 250 μg of anti-mouse CD4 antibody (clone: GK1.5) or an isotype control antibody every 5 days for 15 days. At 28 dpi, ankle joint-associated tissue was analyzed by RT-qPCR or flow cytometry. **(J)** Representative flow cytometry plots of CD4^+^ and CD8^+^ cells among live, singlet, CD45^+^F4/80^−^TCRβ^+^ cells. (**K-L**) Frequency and number of CD4^+^ and CD8^+^ T cells. (**M**) Representative flow cytometry plots depicting CD11c^low^ and MHC-II^+^ cells among live, singlet, CD45^+^F4/80^−^TCRβ^+^CD11c^low^ cells. (**N**) Frequency, number, and gMFI of MHC-II^+^ cells among live, singlet, CD45^+^F4/80^−^TCRβ^+^CD11c^low^ cells. (**O**) RT-qPCR analysis of gene expression. Data are normalized to GAPDH mRNA levels and are expressed as the relative expression (n-fold increase) over expression mock-inoculated mice. Data are representative of 2 independent experiments. *P* values were determined by one-way ANOVA with Tukey’s multiple comparison test (D, H-O) or unpaired Student’s t test (E-G). *, *P*<0.05; **, *P*<0.01; ***, *P*<0.001, ****, *P*<0.0001.

MHC-II expression by macrophages can be induced by IFN-γ and can, in turn, promote IFN-γ production by CD4^+^ T cells.^70^ Given our scRNA-seq and flow cytometry data showing macrophages in the joints of CHIKV-infected mice display elevated expression of MHC-II as other IFN-γ regulated genes, we quantified cells in the joint that expressed *Ifng*. Among all cells present in our scRNA-seq dataset, elevated expression of *Ifng* was detected predominantly within CD4^+^ T cells (Figure 4A–C). Next, we analyzed the top genes expressed in CD4^+^ and CD8^+^ T cells (Figure S3A) and then further divided *Cd3e* expressing cells into clusters of naïve and effector (eff) CD4^+^ and CD8^+^, regulatory (Treg), γδ (Tgd), and unassigned T cells (Figure 4B and Figure S3B). Compared with uninfected control mice, the number of CD4^+^ T_eff_ (18-fold) and CD8^+^ T_eff_ (5.5-fold) cells were increased in joint-associated tissue of CHIKV-infected mice (Figure 4B). Both CD4^+^ and CD8^+^ T_eff_ cells exhibited increased levels of *Ifng* transcripts compared with T_eff_ cells in uninfected control mice and other T cell populations in joint tissue of CHIKV-infected mice (Figure 4C). To confirm these observations, we evaluated *Ifng* expression in cell populations sorted from joint tissue of uninfected control and CHIKV-infected mice at 28 dpi (Figure 4D). This analysis revealed that CD4^+^ T cells express >200-fold greater *Ifng* compared to macrophages and all other cells and CD4^+^ T cells from uninfected control mice, supporting a functional interaction between joint tissue macrophages and CD4^+^ T cells during chronic CHIKV infection.

To further evaluate T cells in joint-associated tissue of control and CHIKV-infected mice during the chronic stage of infection, we performed flow cytometric analysis at 28 dpi. The frequency and number of CD4^+^ T cells in CHIKV-infected mice was >6x greater than those in uninfected control mice (Figure 4E–F). The number of CD8^+^ T cells, but not the frequency, also was increased in joint tissue from CHIKV-infected mice (Figure 4G).

Patients suffering from chronic CHIKV disease exhibit circulating CHIKV specific CD4^+^ T cell responses, primarily targeting viral E1 and nsP1 proteins.^71^ To assess the local CD4^+^ T cell response, we isolated joint-associated cells at 28 dpi from uninfected control mice and mice infected with a recombinant CHIKV strain encoding a CD8^+^ T cell receptor epitope from ovalbumin (OVA).^72^ Stimulation with overlapping CHIKV E1 peptides triggered responses in CD4^+^ T cells, confirming antigen specificity (Figure 4H), while CD8^+^ T cells responded only to the OVA-257 epitope (Figure 4I). There was no effect on the frequency or number of CD4^+^ or CD8^+^ T cells after restimulation (Figure S4A–C). These data suggest either persistent CHIKV antigen presentation is maintained or that antigen-specific CD4^+^ and CD8^+^ T cells are maintained in joint tissue independent of antigen presentation.

CD4^+^ T cells contribute to joint inflammation and swelling during acute CHIKV infection of mice,^39,46,53,73^ but their role in chronic disease is less understood. We found that during chronic infection, CD4^+^ T cells express elevated *Ifng* transcripts in joint tissue (Figure 4A–D), implicating them in sustained inflammation. Moreover, our scRNA-seq analysis revealed that CD4^+^ T cells displayed a transcriptional program suggestive of cell-cell interactions. For example, laminin (*Lmna*), a cytoskeletal protein critical for immunological synapse formation,^74^ was among the top upregulated genes in CD4^+^ T cells of CHIKV-infected mice (Figure S3A), suggesting that joint tissue CD4^+^ T cells actively engage with antigen-presenting cells such as macrophages. To investigate their role, we depleted CD4^+^ T cells starting at 14 dpi. By 28 dpi, flow cytometry confirmed effective depletion in both joint tissue and spleen, with no impact on CD8^+^ T cells or total CD45^+^ cell numbers (Figure 4J–L and Figure S4D–E). While macrophage numbers remained unchanged (Figure S4H–K), the frequency, number, and gMFI of MHC-II^+^ macrophages were significantly reduced following CD4^+^ T cell depletion (Figure 4M–N), suggesting CD4^+^ T cells help maintain MHC-II^+^ macrophages. Further analysis showed that depletion of CD4^+^ T cells reduced *Ifng* and *H2-Aa* (MHC-II) expression, but unexpectedly increased expression of *Il1b* (Figure 4O). Since IFN-γ can suppress *Il1b* transcription,^75,76^ these data suggest distinct regulatory mechanisms are involved in different aspects of chronic joint inflammation following CHIKV infection.

### Joint macrophages harbor viral RNA at the chronic stage of MAYV and RRV infection

**Figure 5.**
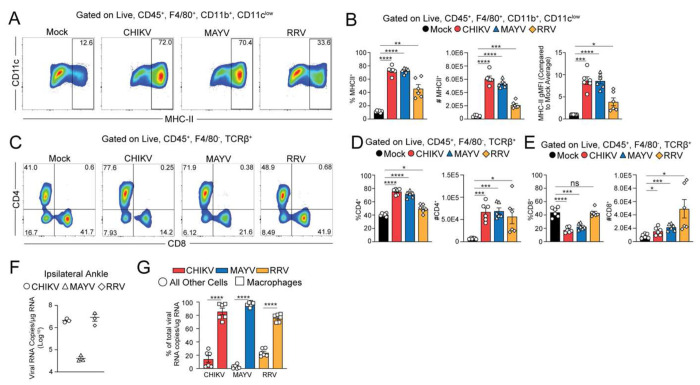
Joint macrophages harbor viral RNA during the chronic stage of MAYV and RRV infection. (**A-D**) WT C57BL/6 mice were inoculated with PBS (mock; n=6) or 10^3^ PFU CHIKV (n=6), MAYV (n=6), or RRV (n=6) in the left rear footpad. At 28 dpi, ankle joint-associated single cells were assessed by flow cytometry. (**A**) Representative flow cytometry plots of CD11c^low^ and MHC-II^+^ cells among live, singlet, CD45^+^F4/80^−^ TCRβ^+^CD11c^low^ cells. (**B**) Frequency, number, and gMFI of MHC-II^+^ cells among live, singlet, CD45^+^F4/80^+^CD11b^+^CD11c^low^ cells. (**C**) Representative flow cytometry plots of CD4^+^ and CD8^+^ cells among live, singlet, CD45^+^F4/80^−^TCRβ^+^ cells. (**D-E**) Frequency and number of CD4^+^ and CD8^+^ T cell populations among live, singles, CD45^+^F4/80^−^TCRβ^+^ cells. (**F-G**) WT C57BL/6 mice were inoculated with PBS (mock) or 10^3^ PFU CHIKV, MAYV, or RRV in the left rear footpad. At 28 dpi, ankle tissue was homogenized in Trizol (n=3/group), or joint-associated single cells were isolated, stained for flow cytometry, fixed, sorted, and viral RNA was quantified by RT-qPCR (n=6/group). Viral RNA copies in **(F)** whole ankle tissue or **(G)** sorted macrophages and all other cells. Data is representative of 2 experiments. *P* values were determined by one-way ANOVA with Tukey’s multiple comparison test (B, D, E) or students t test (G). *, *P* < 0.05; **; ***, *P* < 0.001; ****, *P* < 0.0001.

Given that chronic musculoskeletal disease is associated with infection by a number of different arthritogenic alphaviruses,^2^ we evaluated if mice infected with MAYV or RRV also have elevated MHC-II^+^ macrophages and CD4^+^ T cells in joint tissue during the chronic phase, and if joint macrophages harbor MAYV and/or RRV RNA. Similar to CHIKV-infected mice, at 28 dpi the frequency and number of MHC-II^+^F4/80^+^CD11b^+^CD11c^low^ macrophages, as well as the gMFI of MHC-II on macrophages, were elevated in joint-associated tissue of MAYV- and RRV infected mice compared with PBS-inoculated control mice (Figure 5A–B and Figure S5A–E). In addition, the frequency and number of CD4^+^ T cells were also increased in joint tissue of MAYV-, and RRV-infected mice similar to CHIKV-infected mice (Figure 5C–D). Notably, while the frequency of CD8^+^ T cells in joint tissue was diminished in CHIKV- and MAYV-infected mice compared with mock-infected control mice, this was not observed in RRV-infected mice. Nevertheless, all virus-infected mice displayed an increased number of CD8^+^ T cells in joint tissue (Figure 5D). In all virus infections, the elevated number of MHC-II^+^ macrophages and T cells was associated with the presence of viral RNA in joint-associated tissue (Figure 5F). To determine if this persistent MAYV and RRV RNA is also in macrophages, we sorted macrophages and all other cell types from joint-associated tissue of CHIKV-, MAYV-, and RRV-infected mice at 28 dpi (Figure S5G) and quantified viral RNA in each sorted cell population. In each case, viral RNA was predominantly detected in macrophages (Figure 5G), suggesting that these cells serve as a key reservoir for arthritogenic alphaviruses.

### Joint tissue cells harboring persistent viral RNA are closely localized during chronic CHIKV disease

**Figure 6.**
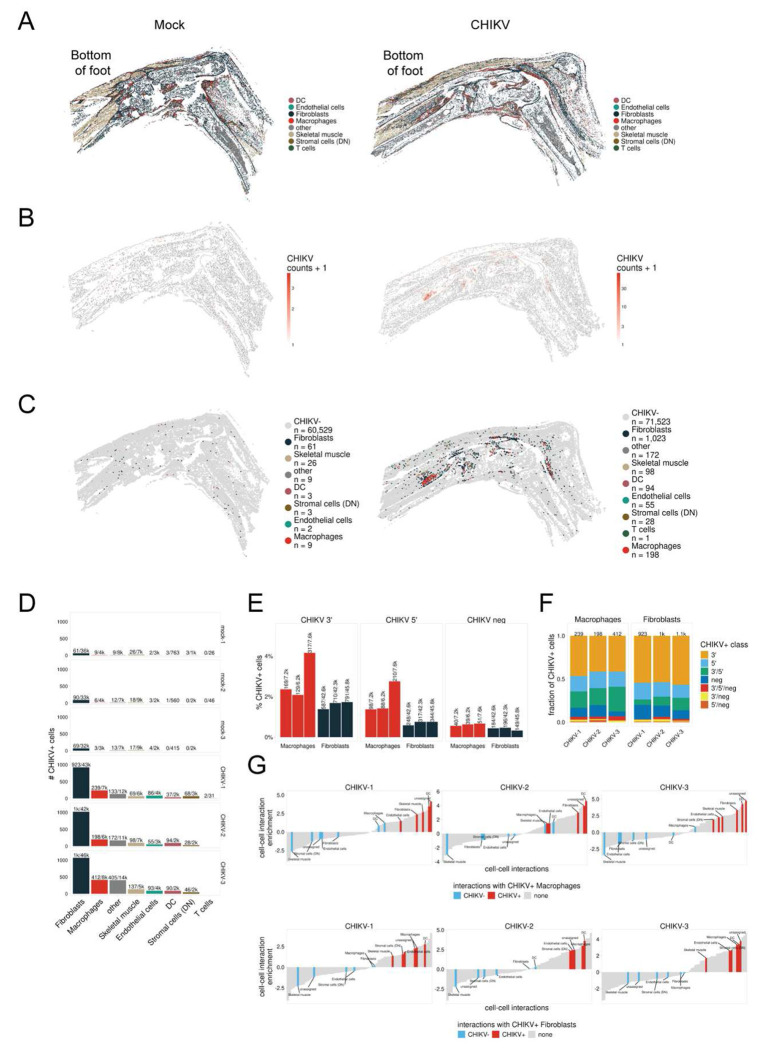
Spatial transcriptomic analysis of joint-associated tissue (**A-G**) WT C57BL/6 mice were inoculated with PBS (mock; n=3) or 10^3^ PFU CHIKV (n=3) in the left rear footpad. At 28 dpi, joint-associated tissue was analyzed by spatial transcriptomics. (**A**) Annotated cell types in mock- and CHIKV-infected joint-associated tissue. (**B**) CHIKV RNA counts in cells of mock- and CHIKV-infected joint-associated tissue. (**C**) Annotated cell types that are CHIKV RNA^+^. (**D**) The number of CHIKV RNA^+^ cells per cell type in each replicate. (**E**) The percent of macrophages and fibroblasts that were positive for 3’ positive-sense RNA, 5’ positive-sense RNA, and negative-strand RNA CHIKV-specific probes. (**F**) Bar graphs showing the fraction of CHIKV RNA^+^ macrophages and fibroblasts that were positive for 3’ RNA positive-sense, 5’ RNA positive-sense, negative-strand RNA, or some combination of CHIKV-specific probes. (**G**) Cell proximity enrichment maps where each highlighted bar represents a cell type that showed increased (positive enrichment) or decreased (negative enrichment) with CHIKV RNA^+^ macrophages (upper) or fibroblasts (lower). CHIKV RNA^−^ cells are annotated in blue while CHIKV RNA^+^ cells are red.

To determine the spatial organization of CHIKV RNA^+^ cells within joint associated tissue during the chronic stage, we analyzed ankle and foot tissue of mock- and CHIKV-infected mice at 28 dpi by virus-inclusive spatial transcriptomics (Figure 6A–G and Figure S6–8). We confidently annotated dendritic cells, endothelial cells, fibroblasts, macrophages, skeletal muscle cells, stromal cells, and a number of other cell types with a prediction confidence score above 0.5 (Figures 6A, S6, and S7). In tissues from CHIKV-infected mice, we detected numerous cells that had between 1-150 CHIKV RNA counts (Figure 6B and Figure S8A). The CHIKV RNA^+^ cells were predominately comprised of fibroblasts and macrophages (Figure 6C–D and Figure S8B). Moreover, across all replicates, we found that the CHIKV signal in both macrophages and fibroblasts was higher for probes that targeted sequences in the 3’ end of the viral genome (Figure 6E), which are also found in the viral subgenomic mRNA produced in infected cells. In addition, we detected both positive-sense and negative-sense CHIKV RNA, in some cases in the same cells (Figure 6E–F), further suggesting active CHIKV RNA replication. Finally, using cell proximity analysis (*see* methods), we identified a positive association between CHIKV RNA^+^ macrophages and fibroblasts and other CHIKV RNA^+^ cells, suggesting these cell types sustain CHIKV RNA in joint-associated tissue during chronic disease (Figure 6G), possibly via cell-to-cell spread which has been reported to occur during CHIKV infection *in vivo* even in the presence of neutralizing antibodies.^77^ Thus, joint-associated CHIKV RNA^+^ fibroblasts and macrophages are highly localized with each other and maintain viral RNA replication during chronic disease.

### CHIKV RNA replication promotes inflammation in joint-associated tissue during chronic disease.

**Figure 7.**
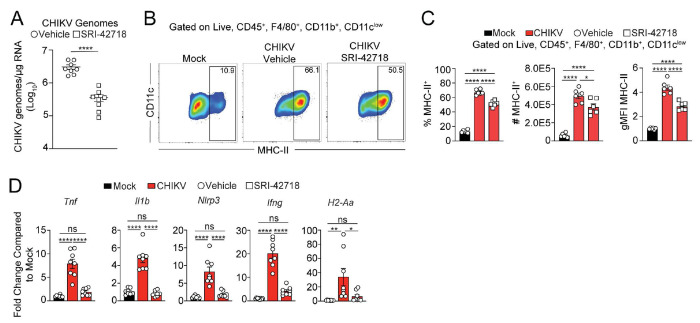
Antiviral therapy initiated during the chronic stage reduced CHIKV RNA and inflammatory gene expression. (**A-D**) WT C57BL/6 mice were inoculated with PBS (mock; n=8) or 10^3^ PFU CHIKV (n=8) in the left rear footpad. At 28 dpi, mice were administered 60 mg/kg of a small molecule antiviral, SRI-42718, or vehicle by oral gavage every 12 h for 7 days. At 35 dpi, ankle joint-associated tissue was isolated for analysis by flow cytometry and RT-qPCR. (**A**) CHIKV RNA copies quantified by RT-qPCR. (**B**) Representative flow cytometry plots of CD11c^low^ and MHC-II^+^ cells among live, singlet, CD45^+^F4/80^−^TCRβ^+^CD11c^low^ cells. (**C**) Frequency, number, and gMFI of MHC-II^+^ cells among live, singlet, CD45^+^F4/80^+^CD11b^+^CD11c^low^ cells. (**D**) RT-qPCR analysis of gene expression. Data are normalized to GAPDH mRNA levels and are expressed as the relative expression (n-fold increase) over expression in mock-inoculated mice. Data are representative of 2 experiments. *P* values were determined by unpaired Student’s t test (A) or one-way ANOVA with Tukey’s multiple comparison test (B-D). *, *P* < 0.05; **, *P* < 0.01; ****, *P* < 0.0001.

Our deep sequencing and spatial transcriptomics data suggests replicating CHIKV RNA persists in joint tissue fibroblasts and macrophages (Figure 6B–F). To test if a direct-acting antiviral can reduce tissue levels of viral RNA and mitigate joint inflammation when administered during the chronic phase, we treated mice with a small molecule inhibitor of alphavirus replication. At 28 dpi, we administered vehicle only or 60 mg/kg of inhibitor every 12 hours for 7 days through oral gavage. At 35 dpi, mice treated with the inhibitor had reduced CHIKV RNA burden in joint associated tissue compared with vehicle-treated mice (Figure 7A), further suggesting that CHIKV RNA persists by a continuous replication mechanism. Although mice treated with the inhibitor showed little to no differences in the number or frequency of macrophages or T cells present in joint-associated tissue (Figure S9), inhibitor treated mice had reduced MHC-II expression on macrophages (Figure 7B–C). Importantly, mice treated with the inhibitor had reduced inflammatory gene expression in joint-associated tissue compared with vehicle treated mice. Indeed, inflammatory gene expression in joints of CHIKV-infected mice treated with the inhibitor was similar to that detected in mock-infected control mice (Figure 7D). These data suggest that viral RNA replication occurs during chronic disease and contributes to joint tissue inflammation, and that targeting the viral RNA replication with a small molecule inhibitor can reduce inflammation.

## DISCUSSION

In this study, we defined cell types that harbor viral RNA and contribute to joint tissue inflammation during the chronic stage of arthritogenic alphavirus infection. Our results suggest that joint tissue macrophages and fibroblasts are major cellular sites of viral RNA persistence, including both positive- and negative-strand viral RNA, and that macrophages are key drivers of chronic joint tissue inflammation. Moreover, treatment of mice with a small molecule inhibitor of alphavirus replication during the chronic stage not only reduced viral RNA levels in joint tissue but also mitigated the inflammatory response. Together, these findings suggest that the persistence of viral RNA in joint macrophages and fibroblasts is maintained by an active viral replicase, and that viral RNA is a trigger of chronic inflammation.

Our scRNA-seq analysis of joint tissue cells at day 28 post-CHIKV infection revealed a gene expression pattern in macrophages with similarity to other inflammatory arthritides, including RA. These findings are consistent with previous studies of clinical, laboratory, and immunological features in human patients, including those with chronic symptoms, indicating that CHIKV-related arthritis resembles seronegative RA.^28^ In addition, prior microarray analysis of gene expression in the feet of CHIKV-infected mice during the acute stage also identified a gene expression pattern with significant similarity to that observed in humans with RA.^78^

Among the cell types identified in our analysis, we found that macrophages and fibroblasts had the largest number of DEGs compared with cells from control mice, while many other cell types exhibited far fewer DEGs. Indeed, all subsets of macrophages identified in the joint (i.e., AQP1^+^, RELMα^+^, lining, and infiltrating) displayed elevated expression of numerous inflammatory genes and MHC-II associated genes, and many of these same genes were identified in prior analyses of gene expression in the joint-associated tissue of CHIKV-infected mice during the acute and chronic stages.^40,78^ Corroborating these findings, we found that macrophages sorted from joint-associated tissue at day 28 post-CHIKV infection displayed elevated levels of inflammatory gene expression, including *Il1b, Tnf, Nlrp3 and H2-Aa*, compared with other cell types from infected mice and with macrophages sorted from joint-associated tissue of uninfected control mice. This is consistent with prior bulk RNA-seq analysis of foot tissue from CHIKV-infected mice at day 30 post infection that also detected an ongoing inflammatory response including elevated *Ifng*, *Il1b*, *Tnf*, *Nlrp3*, numerous chemokines, and immunoproteasome genes.^40^

The numbers of total macrophages and MHC class II^+^ macrophages in joint-associated tissues were elevated during the chronic stage, not only in mice infected with CHIKV but also in mice infected with either MAYV or RRV. Importantly, an increased number of macrophages also were identified in synovial and muscle tissue biopsies of patients with chronic chikungunya or chronic RRV-associated arthritis.^32,33,79^ Collectively, these findings suggest that macrophages are critical drivers of joint inflammation during chronic chikungunya and other chronic forms of alphavirus-induced arthritis. Notably, studies during the acute stage of CHIKV infection in mice also identified the accumulation of MHC class II^+^ macrophages in joint-associated tissue, and depletion of these cells ameliorated acute joint pathology.^18,46^ We found that day 28 joint tissue macrophages in CHIKV-infected mice expressed elevated levels of *Nlrp3* and *Il1b*. Prior transcriptional analysis of PBMCs from patients with acute CHIKV infection identified activation of the NLRP3 inflammasome as a key feature of CHIKV infection.^80^ In addition, exposure of murine bone marrow macrophages to CHIKV or MAYV induced IL-1β production by a mechanism that was dependent on NLRP3 inflammasome activation,^80,81^ and inhibition of NLRP3 or *Nlrp3* deficiency reduced CHIKV and MAYV induced inflammation during acute infection in mice.^45,81^ Collectively, these data further highlight that there are overlapping features of acute and chronic joint inflammation following alphavirus infection and suggest that sustained viral activation of the NLRP3 inflammasome in joint tissue macrophages may promote chronic disease.

In addition to macrophages, CD4^+^ T cells also have been identified in synovial and muscle tissue biopsies of patients with chronic chikungunya,^32,33^ and studies in mice implicated these cells as mediators of joint pathology during acute CHIKV infection.^39,46,53,73^ Recently, analysis of human PBMCs collected from chikungunya patients years after infection found that CHIKV-specific CD4^+^ T cell responses, particularly against epitopes in nsP1 and E1, were significantly stronger in patients with chronic disease compared with those in which symptoms had resolved.^71^ Using this same E1 peptide pool, we identified E1-specific CD4^+^ T cells in the joints of CHIKV-infected mice during the chronic stage. In addition, our scRNA-seq analysis found that the number of CD4^+^ T_eff_ cells was increased and these cells expressed elevated *Ifng*, which was confirmed by RT-qPCR analysis of sorted CD4^+^ T cells. Moreover, depletion of CD4^+^ T cells reduced MHC class II expression on joint tissue macrophages, suggesting a functional interaction between these cells. Similar macrophage-CD4^+^ T cell interactions were observed in mice during acute CHIKV infection,^46^ further highlighting that there are overlapping features of acute and chronic joint inflammation following CHIKV infection.

The role of persistent infection or the persistence of viral products in chronic alphavirus-induced arthritis in humans remains to be determined. While a few studies have detected viral RNA and/or antigen in musculoskeletal tissues of patients with chronic alphavirus arthritis,^32,33,79^ other studies have reported negative results.^34,35^ Nevertheless, numerous studies suggest that alphaviruses are capable of establishing persistent infection. Indeed, long-term persistence of viral RNA but not infectious virus, was detected in the brain of mice following Sindbis virus and Semliki Forest virus infection, and this viral RNA supported a resurgence of infectious virus production when immune control was blocked.^82,83^ In addition, noncytopathic, persistent alphavirus RNA replication can be observed in mammalian cells *in vitro*.^84–87^ Our studies identified joint tissue macrophages and fibroblasts as the predominant cellular sites of CHIKV RNA persistence. Importantly, deep sequencing analysis of viral RNA present in joint tissue and joint tissue macrophages during the chronic stage detected reads spanning the full-length genome. Moreover, our spatial transcriptomics analysis revealed the presence of both positive-sense and negative-sense CHIKV RNA, with fibroblasts and macrophages containing the highest proportion of cells exhibiting this viral RNA replication signature. These findings suggest that these cell types serve as sites of persistent viral replication and their close proximity may support cell-cell viral spread. In support of this idea, therapeutic treatment of mice with a small molecule inhibitor of viral replication reduced the persistent CHIKV RNA in joint-associated tissue. Critically, the reduced viral RNA in mice that received antiviral treatment was associated with decreased MHC-II expression on macrophages and pro-inflammatory gene expression. These data provide compelling evidence that CHIKV RNA replication in macrophages and fibroblasts promotes joint tissue inflammation and supports the hypothesis that antiviral therapy could mitigate chronic disease severity.

### Limitations of the study

A cell type pertinent to CHIKV pathogenesis is skeletal muscle fibers,^43,88,89^ which are typically lost when preparing single cell suspensions for analysis by flow cytometry or scRNA-seq. Thus, many of our analyses did not capture information for this important cell type. Nevertheless, we did detect skeletal muscle cells by spatial transcriptomics analysis of foot and ankle tissue and found only small numbers of these cells were CHIKV RNA^+^, suggesting that skeletal muscle cells may not be a major cellular site of viral RNA persistence. However, further work is needed to confirm these findings. Our deep sequencing analysis of the CHIKV genome in joint tissue and isolated joint tissue macrophages suggests that adaptive mutations are not required for persistence of viral RNA at these sites. Similarly, a prior RNA-seq study of CHIKV-infected mouse feet at day 30 did not detect significant viral sequence variants.^40^ However, each of these analyses were performed at a single time point and it remains possible that adaptive mutations would become enriched in the viral genome over longer periods of time. Finally, while the mouse model of chronic chikungunya used in this study recapitulates many of the hallmarks of chronic chikungunya in humans, the model has some limitations including lower levels of persistent viral RNA and inflammation in joint tissues that are distal to the site of inoculation and the absence of a relapsing/remitting disease pattern observed in some chronic chikungunya patients.^30^

## METHODS

### Ethics statement.

This study was conducted in accordance with the recommendations in the Guide for the Care and Use of Laboratory Animals and the American Veterinary Medical Association (AVMA) Guidelines for the Euthanasia of Animals. All animal experiments conducted at the University of Colorado Anschutz Medical Campus were performed with the approval of the Institutional Animal Care and Use Committee (IACUC) of the University of Colorado School of Medicine (Assurance Number: A3269-01) under protocols 00026 and 00215. Experimental animals were humanely euthanized at defined endpoints by exposure to isoflurane vapors followed by bilateral thoracotomy.

### Viruses.

CHIKV AF15561, MAYV CH (provided by Scott Weaver, University of Texas Medical Branch), and RRV T48 were generated from cDNA clones as described previously.^17,41,90^ CHIKV-VENKL, which encodes the CD8^+^ TCR epitope SIINFEKL, as generated as previously described.^51^ Briefly, plasmids were linearized by NotI, PacI, or SacI digestion and used as a template for *in vitro* transcription with SP6 DNA-dependent RNA polymerase (Ambion). Capped RNAs were concentrated by lithium chloride precipitation, evaluated by gel electrophoresis, and then electroporated into BHK-21 cells. At 24-28 hours post electroporation, cell culture supernatants were collected and clarified by centrifugation. Clarified supernatants were aliquoted and stored at −80°C and virus titers were determined by plaque assay.

### Mouse experiments.

WT C57BL/6J mice were obtained from The Jackson Laboratory. All experiments were performed in 4-week-old male mice. Mice were anesthetized with isoflurane vapors and inoculated in the left-rear footpad with a 10 μL volume containing 10^3^ PFU of virus diluted in phosphate buffered saline (PBS)/1% fetal bovine serum (FBS) using a Hamilton syringe and 30G needle. Following euthanasia, blood was collected, and mice were intracardially perfused with 10 mL of PBS at the indicated time points. To deplete CD4^+^ T cells, mice were intraperitoneally (IP) inoculated with 250 μg of either isotype control antibody (LTF-2; Bioxcell) or anti-CD4 antibody (GK1.5; Bioxcell) diluted in PBS to a final volume of 200 μL, every 5 days starting at 14 dpi. Mice were harvested at 28 dpi. Depletion efficiency was determined by flow cytometric analysis of ankle and spleen tissue. To inhibit CHIKV RNA replication *in vivo*, at 28 dpi mice were administered vehicle only (10% 1-Methyl-2-Pyrrolidone (NMP), 30% saline, and 60% polyethylene glycol (PEG) or 60 mg/kg of SRI-42718 in vehicle by oral gavage every 12 hours for 7 days. At 35 dpi, tissues were collected and analyzed as outlined below.

### Preparation of joint tissue single-cell suspensions.

Single cell suspensions were generated from joint-associated tissues by mechanical and enzymatic digestion as previously described.^50,51^ At the indicated time points, the ipsilateral ankle and foot tissue from mock- or virus-inoculated mice were dissected. Tissues were placed in a 15 mL conical containing five 1.0 mm glass beads and horizontally agitated in RPMI 1640 (Gibco) medium supplemented with 10% fetal bovine serum (FBS), 15 mM HEPES, 2.5 mg/mL collagenase A (Roche), and 1.7 mg/mL DNase I (Sigma) for 2 h at 37°C. After incubation, digested tissues were filtered by passing through a 70 μm cell strainer, cells were washed in wash buffer (1X Hanks balanced salt solution, 15 mM HEPES), and total viable cells were determined by trypan blue exclusion.

#### Single cell mRNA sequencing

##### Single-cell library preparation using the 10X Genomics platform.

We targeted recovery of 10,000 cells for scRNA-seq for each sample. Final cell suspensions were emulsified, lysed, and barcoded using the Next GEM Chip G Kit (1000127) and a 10X Genomics chromium controller housed in a BSL3 laboratory. Single-cell gene expression libraries were generated using the Next GEM single-cell 3′ GEM library and gel bead kit v3.1 (1000128) and single index kit T set A (1000213) according to the manufacturer’s instructions (10× Genomics). Sequences were generated with the Illumina NovaSEQ 6000 instrument using S4 flow cells and 300 cycle SBS reagents. We targeted 50,000 reads per cell, with sequencing parameters of Read 1:151 cycles; i7 index: 8 cycles; i5 index: 0 cycles; Read 2: 151 cycles.

##### CHIKV-capture library preparation.

The scRNA-seq libraries were enriched for molecules aligning to the CHIKV genome according to our previously published methods.^91^ Specifically, the CHIKV genome was PCR amplified in 3 fragments (primer sequences: CHIKV-F1 5’ – TGAGACACACGTAGCCTACCA – 3’, CHIKV-F2 5ʹ - AAGTCCAAGGGAATACAGATCTTC – 3’, CHIKV-F3 5’ – ACCGCAGCACGGTAGAGA – 3’, CHIKV-R1 5’ – CGAATAACATTACCTTGGAGCA – 3’, CHIKV-R2 5’ – TTTTTCCCGGCCTATCACAG – 3’, CHIKV-R3 5’ – AAAAACAAAATAACATCTCCTACGTC – 3’) and labeled with biotin-dUTP using the same primers before sonicating to generate ~150 bp fragments for hybridization. Denatured and diluted biotin-dUTP-labeled CHIKV genome fragments were hybridized to the concentrated scRNA-seq libraries separately. Streptavidin capture beads were mixed with the hybridized libraries and washed to remove unbound DNA. Libraries were amplified directly from the cleaned-up beads and sequenced as described above. FASTQ files for each replicate (3 mock- and CHIKV-infected) were processed using the cellranger count pipeline (v6.0.1).

##### scRNA-seq gene expression analysis.

FASTQ files for each replicate (3 mock- and 3 CHIKV-infected) were processed using the cellranger count pipeline (v6.0.1). Reads were aligned to a version of the mm10 reference genome that also included the CHIKV AF15561 (EF452493.1) genome. The CHIKV genome included annotations for the sgRNA (position 7567-12036) and 5’ (position 1-7566) regions. Initial filtering of gene expression data was performed separately for each biological replicate using the Seurat R package (v4.3.1). Gene expression data for each biological replicate were combined into a single Seurat object. CHIKV counts were excluded from the gene expression matrices so they would not influence downstream processing (dimensionality reduction, clustering) of the mouse expression data. Cells were filtered based on the number of detected mouse genes (>250 and <5,500) and the percent mitochondrial counts (<30%). Genes were filtered to only include those detected in >5 cells. Potential cell doublets were removed using the DoubletFinder (v2.0.3) R package. Mouse gene expression counts were normalized by the total mouse counts for the cell, multiplied by a scale factor (10,000), and log-transformed (NormalizeData). The gene expression data for each biological replicate were combined into a single Seurat object. The normalized mouse counts were scaled and centered (ScaleData) using the top variable features identified with the M3Drop (v1.28.0) R package. The scaled data were used for PCA (RunPCA), and the first 50 principal components were used to identify clusters (FindNeighbors, FindClusters) and calculate uniform manifold approximation and projection (UMAP) (RunUMAP). Counts from the CHIKV-capture libraries were then added to the object for all cells passing our filtering cutoffs. CHIKV^+^ cells were classified as any cell with at least 1 CHIKV RNA count in either the CHIKV-capture or bulk scRNA-seq libraries.

We generated an initial set of broad cell type annotations using the R package, clustifyr^92^ (v1.14.0) and reference data from Immgen.^93^ These annotations were checked for accuracy and further refined using known cell type markers. To annotate macrophage subsets, macrophages were re-clustered, and samples were integrated using the R package, Harmony (v1.2.0).^94^ Macrophage clusters were annotated using published marker genes.^58^ To annotate T cell subsets, T cells were re-clustered, samples were integrated using the Harmony package, and clusters were annotated using known marker genes.

Differentially expressed genes were identified for each cell subset for mock vs CHIKV-infected samples using the Seurat R package. P values were adjusted for multiple testing using the Benjamini-Hochberg method and differentially expressed genes were filtered to only include those with an absolute log2 fold change >0.25 for all three replicates, a maximum adjusted p value <0.05. Mitochondrial genes were excluded. Differentially expressed genes were identified for all macrophages and T cell subsets from mock vs CHIKV-infected samples using the Seurat package. Due to the low number of T cells, all replicates were grouped together. Gene set enrichment analysis (GSEA) was performed using the clusterProfiler (v4.10.0) R package, GSEA terms were filtered to remove broad terms that include >400 genes.

##### RNA isolation and RT-qPCR quantification of RNA from joint-associated tissue.

To quantify gene expression and viral RNA in tissues, ankle tissue was dissected from mice, directly added to Trizol (Life Technologies) with 1.0 mm glass beads (Biospec Products) and homogenized using a FastPrep-24 Classic homogenizer (MP Biomedical). Total RNA was isolated using a PureLink RNA mini kit (Life Technologies) and cDNA was generated using random hexamers and SuperScript IV reverse transcriptase (Life Technologies). CHIKV RNA was quantified by qPCR with CHIKV specific forward (5’-TTTGCGTGCCACTCTGG-3’) and reverse primers (5’-CGGGTCACCACAAAGTACAA-3’), with an internal TaqMan probe (5’-ACTTGCTTGATCGCCTTGGTGAGA-3’) all within the nsP2 gene region, as previously described.^50^ MAYV RNA was quantified using MAYV specific forward (5’-AAGCTCTTCCTCTGCATTGC-3’) and reverse primers (5’-TGCTGGAAACGCTCTCTGTA-3’), with an internal TaqMan probe (5’-GCCGAGAGCCCGTTTTTAAAATCA-3’) within the nsP1 gene region. RRV RNA was amplified through the generation of cDNA with a sequence-tagged (in lowercase letters) RRV specific RT primer (5’-ggcagtatcgtgaattcgatgcAACACTCCCGTCGACAACAGA-3’) and quantified by qPCR with an RRV specific forward primer (5’-CCGTGGCGGGTATTATCAAT-3’) and a tag sequence-specific reverse primer (5’-GGCAGTATCGTGAATTCGATGC-3’), with an internal TaqMan probe (5’-ATTAAGAGTGTAGCCATCC-3’) within the nsP3 gene region. The relative fold of host gene expression between mock- and CHIKV-infected samples was determined using the comparative Ct method^95^ and commercially available RT-qPCR TaqMan Gene Expression Assays (ThermoFisher Scientific): IL-1β (Mm00434229_m1), TNF (Mm00443258_m1), H2-Aa (Mm00439211_m1), IFN-γ (Mm01168134_m1), and NLRP3 (Mm00840904_m1). GAPDH (Mm99999915_g1) was used as a control to normalize for input amounts of cDNA.

##### RNA Isolation and RT-qPCR from fixed-sorted cells.

To sort cells from joint-associated tissue, single cell suspensions were stained for flow cytometry as described above and fixed in 2% paraformaldehyde (PFA)/PBS for 10 min at room temperature. Samples were sorted using a MoFlo XDP100 (Beckman Coulter) flow cytometer. RNA was extracted from fixed-sorted cells with the RNeasy FFPE Kit (Qiagen) and cDNA was generated using random hexamers and SuperScript IV reverse transcriptase (Life Technologies 18091200). CHIKV, MAYV, or RRV nucleic acid and host gene copies were quantified using methods described above.

#### Deep sequencing of CHIKV RNA.

##### Sample Sequencing.

Viral whole genome sequencing (WGS) of CHIKV inoculum from viral stocks was performed using metagenomic next-generation sequencing (mNGS) protocol as described previously.^96^ Briefly, extracted RNA was treated with a TURBO DNA-free Kit (Thermo Fisher, AM1907), reverse transcribed using random hexamers (Thermo Fisher, N8080127) and Superscript IV (Thermo Fisher, 18090010) according to the manufacturer’s protocol. Double-stranded cDNA synthesis was performed using Sequenase v2.0 (Thermo Fisher, 70775Z1000UN), and the resulting cDNA was purified using 1.8x AMPure XP magnetic beads (Beckman Coulter, A63882). Metagenomic viral WGS libraries were created from purified double-stranded cDNA with tagmentation reagents from the Illumina DNA Prep with Enrichment Kit (Illumina, 20060059), followed by 18 cycles of dual-indexed PCR. Amplified libraries were cleaned with 0.8x AMPure XP magnetic beads. Viral isolate libraries were not subjected to enrichment/hybridization capture due to high viral content. Viral WGS was also performed on three joint-associated tissues and three sorted joint tissue macrophage specimens with high viral RNA loads, as determined by RNA copies per μg of total RNA. These samples were processed using QIAseq xHYB Microbial Hyb&Lib Kit A (Qiagen, 334525) and a custom Qiagen hybridization panel synthesized based on CHIKV strain AF15561 (EF452493.1) following the manufacturer’s protocol. In short, extracted RNA was depleted of mouse ribosomal RNA, converted to double-stranded cDNA, enzymatically fragmented, end-repaired, indexed, purified using 0.9x and 1.1x bead cleanups, and amplified with 14 cycles of PCR followed by a final bead purification. Pre-capture libraries were then pooled based on RNA copies/μg RNA (one to three samples per pool) and hybridized overnight with a custom biotinylated probe panel. Probe-bound targets were captured using streptavidin-coated magnetic beads and washed to remove non-specific fragments. Enriched libraries were amplified with 20 cycles of PCR and purified using a 1.1x bead cleanup. Final library concentrations were quantified with the Qubit 4 Fluorometer and dsDNA HS Assay Kit. All libraries were sequenced on Illumina NextSeq 2000 instruments using a 2x150bp read format.

##### Data analysis.

Raw sequencing reads were trimmed and quality-filtered using fastp (v0.23.4) with the following settings: --cut_mean_quality 20 --cut_front --cut_tail --length_required 20 --low_complexity_filter --trim_poly_g --trim_poly_x. Quality-controlled reads from the viral inoculum stocks were randomly downsampled to 1 million paired-end reads using seqtk (v1.4) Consensus genomes for the inoculum stocks were generated by majority rule in Geneious Prime 2025.0, using the chikungunya virus strain AF15561 (EF452493.1) as the reference. Variant calling was performed with the RAVA pipeline (default settings), using the matched inoculum consensus as a reference (https://github.com/greninger-lab/RAVA_Pipeline/tree/2024-12-09_CU_KZ_CHIKV_publication)

##### Flow cytometry.

Single cell suspensions were blocked with anti-FcγRIII/II (2.4G2; BD Pharmingen) for 10 min at room temperature, stained with LIVE/DEAD Fixable Violet Dead Cell Stain (Invitrogen) according to product instructions, then stained with the indicated antibodies for 45 min on ice. Cells were washed 2 times with FACS buffer (1% FBS, 2 μM EDTA, 20 mM HEPES in 1x PBS) and fixed by addition of PFA to a final concentration of 1% for 10 min at room temperature. For intracellular stains, fixed cells were incubated with the indicated antibodies in 0.1% saponin in FACS buffer for 0.5-2 h at room temperature, washed 3 times with 0.1% saponin in FACS buffer, and resuspended in FACS buffer. Samples were acquired using a Cytek Aurora cytometer (> 50,000 events) and Aurora software. Downstream analysis was performed using FlowJo software (Tree Star). All antibodies were purchased from BioLegend unless otherwise indicated: CD11b (M1-70), CD11c (N418), TCRβ (H57-597), CD3ε (145-2C11), CD4 (GK1.5), CD8 (53-6.7), MHC-II (IA/IE; M5/114.15.2; eBioscience or BD Biosciences), F4/80 (BM8), CD45 (30-F11; Biolegend or BD Biosciences), NK1.1 (PK136), Ly6G (1A8), Ly6C (HK1.4), B220 (RA3-6B2), IFN-γ (XMG12), CD90.2 (30-H12; Invitrogen).

##### Ex vivo T cell stimulation.

Single cell suspensions of joint-associated tissue collected at 28 dpi were stimulated *ex vivo* with the MHC class I ovalbumin (OVA) peptide, OVA-257 (1 μg), or with an E1 peptide pool^71^ (1 μg) in the presence of 3 μg/mL brefeldin A for 5 h and then processed for surface staining as described above. Following surface staining and PFA fixation, cells were washed two times in saponin buffer (1 mg/mL saponin in FACS buffer). For intracellular stain, cells were resuspended in a cocktail of intracellular antibodies in saponin buffer for 2 hours at RT. After incubation, cells were washed twice with saponin buffer and acquired on the Cytek Aurora cytometer.

#### Spatial Transcriptomics.

##### Tissue Processing.

Paraffin-embedded mouse ankle blocks were sectioned on a microtome under RNAse free conditions to prepare slides for Xenium analysis. 5 micron sections were placed onto Xenium slides (10X Genomics PN-3000941), with 3 animal’s ankles included per slide, and allowed to airdry overnight in a slide desiccator. Slides were then baked for 2 hours at 60C, followed by deparaffinization and decrosslinking. Panels of oligonucleotide probes including the 379 gene Xenium Mouse Tissue Atlassing Panel (10x Genomics PN-2000949) ) and a custom panel of 98 genes (Design ID QX42XM) were hybridized to RNA targets in -situ. Our custom gene panel included targets for the CHIKV genome, with separate probes for 3’ (position 7567-12036) and 5ʹ (position 1-7566) regions, and for negative-strand CHIKV RNA. Rolling circle amplification (RCA) was used to boost fluorescent signal following the steps outlined in Xenium In Situ Gene Expression User Guide CG000749 Rev B. Slides were loaded onto the Xenium Analyzer (10X Genomics PN-1000569) for imaging and analysis. Regions of interest were manually selected, one region per sample, before capture. Cell segmentation staining reagents (PN 1000661) were applied to the tissue as described in User Guide CG000749 Rev B to aid in accurate assignment of cell boundaries during analysis.

##### Data Analysis.

Data for each tissue section was processed as a separate “field of view” (FOV) using the Seurat R package (v4.4.2). For each slide a separate Seurat object was generated for each FOV. CHIKV counts were added to the objects as a separate assay. Gene expression counts for each cell were normalized (SCTransform), and PCA and UMAP were performed (RunPCA, RunUMAP) followed by clustering (FindNeighbors, FindClusters). To annotate cell types, we first generated a cell type reference using our scRNA-seq data for the mock-infected replicate with the greatest number of cells. We then transferred cell type labels to each Xenium FOV object using Seurat (FindTransferAnchors, TransferData). Cells with a cell type prediction confidence <=0.5 were labeled as unassigned. CHIKV^+^ cells were identified as any cell with at least 1 CHIKV count. To assess cell-cell proximity enrichment for CHIKV^+/−^ cells, we used the Giotto R package (v4.2.1).^97^ For each FOV we created a separate Giotto object (createGiottoXeniumObject) containing cell type annotations for CHIKV^+^ and CHIKV^−^ cells. We then created a spatial Delaunay network (createSpatialDelaunayNetwork) and computed cell-cell interaction enrichment (cellProximityEnrichment). Cell types were only included if they had >=20 cells CHIKV^+^ cells for all three biological replicates. Interaction results were filtered to only include interactions with a *P* value <0.05.

### Quantification and statistical analysis.

Flow Cytometry and RT-qPCR data were analyzed using GraphPad Prism version 10.5.0 software. Column heights indicate mean values; error bars indicate the SEM. Boxplots were drawn as follows: center line, median; box limits, upper and lower quartiles; whiskers, 1.5x interquartile range; points, outliers. For statistical analysis, data were evaluated using either one-way analysis of variance (ANOVA) with multiple comparisons test or student’s t-test. *P* values of <0.05 were considered significant. All differences not indicated as significant had *P* values of >0.05. The number of replicates per experiment and the *P* values are indicated in the figure legends.

## Supplementary Material

1

## Data Availability

The authors declare that all data supporting the findings of this study are available within the paper, its Extended Data, or Source Data files. The scRNA-seq and spatial transcriptomics data have been deposited in the NCBI GEO database (GSE300299, GSE300303). Analysis pipelines are available at https://github.com/rnabioco/morrison-chronic-chikv and https://github.com/rnabioco/morrison-chronic-chikv-xenium. An analysis pipeline for the WGS is available at https://github.com/greninger-lab/RAVA_Pipeline/tree/2024-12-09_CU_KZ_CHIKV_publication, and raw sequencing data is available at NCBI BioProject PRJNA1247591 (https://www.ncbi.nlm.nih.gov/bioproject/?term=PRJNA1247591).
