# Supplementary material for: Chikungunya virus persists in joint-associated macrophages and promotes chronic disease
Source: Res Sq. 2025 Jun 25:rs.3.rs-6917990. Preprint. [Version 1] doi: 10.21203/rs.3.rs-6917990/v1 (PMC12270208; doi:10.21203/rs.3.rs-6917990/v1)
Supplement: 1 [file NIHPPrs6917990v1-supplement-1.pdf]

SUPPLEMENTAL FIGURE LEGENDS

Figure S1. Cell sorting strategy for macrophages, CD4$^+$ T cells, and all other cells from joint-associated tissue during chronic CHIKV disease. (**A**) WT C57BL/6 mice were inoculated with PBS (mock; n=6) or 10^3 PFU CHIKV (n=6) in the left rear footpad. At 28 dpi, CHIKV RNA copies in whole ankle homogenates or homogenates of single cell suspensions generated from enzymatically digested ankle tissue were quantified by RT-qPCR. (**B**) Representative flow cytometry plots demonstrating cell gates used to sort designated cell populations as well as the

purity of each sorted cell population. (**C**) The total number of cells sorted for each of the cell populations from mock- (n=6) and CHIKV-infected (n=6) mice. Data are from 2 independent experiments (A, C) while flow plots are representative of 2 independent experiments comprised of 3 replicates each (B).

Figure S2. Characterization of joint macrophages during chronic CHIKV disease. (**A**) WT C57BL/6 mice were inoculated with PBS (mock; n=3) or 10^3 PFU CHIKV (n=3) in the left rear footpad. At 28 dpi, ankle joint-associated single cells were analyzed by scRNA-seq. Shown are heatmaps of the top 10 marker genes for each macrophage subset. (**B**) Coverage maps for the virus inoculum used for Figure 2G-H. (**C**) Coverage maps represented in Figure 2G-H with synonymous (black) or nonsynonymous mutations (blue) above 10% allele frequency indicated. (**D**) UMAPs and bar graphs of expression of selected inflammatory genes in macrophage subsets of mock-and CHIKV-infected mice. **(E)** Representative flow cytometry plots for MHC-II expression on live, singlet, $CD45^+CD11b^+CD11c^{high}$ cells. (**F**) Frequency, number, and gMFI of MHC-II on $CD11c^{high}$ cells. Data are representative of 2 independent experiments. *P* values were determined by unpaired Student's test (E-F). **, *P*<0.01; ****, *P*<0.0001.

Figure S3. scRNA-seq analysis of joint-associated T cells during chronic CHIKV disease. (**A**) Heatmap showing genes upregulated in T cell subsets from CHIKV-infected mice at 28 dpi (m = mock; C = CHIKV). Genes with a grey diamond are significantly upregulated. Genes with a black diamond are significantly upregulated and are within the top 15 upregulated genes for the subset. (**B**) Heatmap showing the expression of the top 10 marker genes for each T cell subset (m= mock; C = CHIKV). ScRNA-seq data are representative from one experiment with 3 mice/group.

Figure S4. $CD4^+$ T cells regulate MHC-II^+ macrophages during chronic disease. (**A-C**) WT C57BL/6 mice were inoculated with PBS (mock; n=6) or 10^3 PFU CHIKV-OVA (n=6) in the left rear footpad. At 28 dpi, ankle joint cells were analyzed by flow cytometry. At 28 dpi, ankle joint single cell suspensions were stimulated *ex vivo* with the indicated peptides for 5 h in the presence of brefeldin A. (**A**) Representative flow cytometry plots of $CD4^+$ and $CD8^+$ cells among live, singlet, $CD45^+F4/80^-TCR\beta^+$ cells. (**B**) Frequency and number of $CD4^+$ T cells in stimulation groups. (**C**) Frequency and number of $CD8^+$ T cells in stimulation groups. (**D-K**) WT C57BL/6 mice were inoculated with PBS (mock; n=6) or 10^3 PFU CHIKV (n=6) in the left rear footpad. At 14 dpi, mice were injected intraperitoneally with 250 µg of anti-mouse CD4 antibody (clone: GK1.5) or an isotype control antibody every 5 days. At 28 dpi, ankle joint-associated tissue and the spleen were analyzed by flow cytometry. (**D**) Representative flow cytometry plots of splenic $CD4^+$ and $CD8^+$ cells among live, singlet, $CD45^+F4/80^-TCR\beta^+$ cells. (**E**) Frequency and number of splenic $CD4^+$ T cells. (**F**) Representative flow cytometry plots of $CD45^+$ cells in joint-associated tissue among live, singlet cells. (**G**) Frequency and number of $CD45^+$ cells among live, singlet cells. (**H**) Representative flow cytometry plots of $F4/80^+$ cells in joint-associated tissue among live, singlet, $CD45^+$ cells. (**I**) Frequency and number of $F4/80^+$ cells in joint-associated tissue among live, singlet, $CD45^+$ cells. (**J**) Representative flow cytometry plots of $CD11b^+$ and $CD11c^+$ cells in joint-associated tissue among live, singlet, $CD45^+$, $F4/80^+$ cells. (**K**) Frequency and number of $CD11c^{low}$ cells in joint-associated tissue among live, singlets, $CD45^+$, $F4/80^+$, $CD11b^+$ cells. Data are representative of 2 independent experiments. *P* values were determined by one-way ANOVA with Tukey's multiple comparison test (B-C, G, I, K) or unpaired Student's t test (E). *, $P < 0.05$; **, $P < 0.01$; ***, $P < 0.001$; ****, $P < 0.0001$.

Figure S5. Macrophages are cellular sites of viral RNA persistence during CHIKV, MAYV, and RRV infection. (**A-F**) WT C57BL/6 mice were inoculated with PBS (mock; n=6) or 10^3 PFU CHIKV (n=6), MAYV (n=6), or RRV (n=6) in the left rear footpad. At 28 dpi, ankle joint-associated

single cells were assessed by flow cytometry. (**A**) Representative flow cytometry plots of CD45$^+$ cells among live, singlet cells. (**B**) Frequency and number of CD45$^+$ cells. (**C**) Representative flow cytometry plots of F4/80$^+$ cells among live, singlet, CD45$^+$ cells. (**D**) Frequency and number of F4/80$^+$ cells. (**E**) Representative flow cytometry plots of CD11b$^+$ and CD11c$^+$ cells among live, singlet, CD45$^+$, F4/80$^+$ cells. (**F**) Frequency and number of CD11c^{low} cells. (**G**) FACS gating strategy for sorting all other cells and macrophages from joint-associated tissue. Data are representative of 2 independent experiments. *P* values were determined by unpaired Student's t test (B, D, F). *, $P < 0.05$; **, $P < 0.01$; ***, $P < 0.001$; ****, $P < 0.0001$.

Figure S6. Spatial transcriptomics: cell type annotation. (**A-B**) WT C57BL/6 mice were inoculated with PBS (mock; n=3) or 10^3 PFU CHIKV (n=3) in the left rear footpad. At 28 dpi, joint-associated tissue was analyzed by spatial transcriptomics. (**A**) Per cell predication confidence for cell type annotations. (**B**) Annotated cell types shown for each tissue section. Bar graphs display the percent of each cell type among total cells and the total number of each cell type detected in each independent tissue section.

Figure S7. Spatial transcriptomics: spatial distribution of cell types. (**A-B**) WT C57BL/6 mice were inoculated with PBS (mock; n=3) or 10^3 PFU CHIKV (n=3) in the left rear footpad. At 28 dpi, joint-associated tissue was analyzed by spatial transcriptomics. (**A**) Representative heatmaps showing the spatial distribution of annotated cell types in tissue from mock-infected mice. (**B**) Representative heatmaps showing the spatial distribution of annotated cell types in tissue from CHIKV-infected mice.

Figure S8. Spatial transcriptomics: CHIKV RNA$^+$ cells. (**A-B**) WT C57BL/6 mice were inoculated with PBS (mock; n=3) or 10^3 PFU CHIKV (n=3) in the left rear footpad. At 28 dpi, joint-associated tissue was analyzed by spatial transcriptomics. (**A**) CHIKV RNA counts in cells of

mock- and CHIKV-infected joint-associated tissue are shown for each tissue section. (**B**) CHIKV RNA$^+$ cells (>0 total CHIKV counts) are shown for each tissue section.

Figure S9. Treatment with a small molecule antiviral during chronic CHIKV disease diminishes MHC-II$^+$ macrophages. (**A-I**) WT C57BL/6 mice were inoculated with PBS (mock; n=8) or 10^3 PFU CHIKV (n=8) in the left rear footpad. At 28 dpi, mice were administered 60 mg/kg of a small molecule antiviral, SRI-42718, by oral gavage every 12 h for 7 days. At 35 dpi, ankle joint-associated tissue was isolated for analysis by flow cytometry. (**A**) Representative flow cytometry plots of CD45$^+$ cells among live, singlets cells. (**B**) Frequency and number of CD45+ cells. (**C**) Representative flow cytometry plots of F4/80$^+$ cells among live, singlet, CD45$^+$ cells. (**D**) Frequency and number of F4/80$^+$ cells. (**E**) Representative flow cytometry plots of CD11b$^+$ and CD11c$^+$ cells among live, singlet, CD45$^+$, F4/80$^+$ cells. (**F**) Frequency and number of CD11b$^+$CD11c^{low} cells among live, singlet, CD45$^+$, F4/80$^+$ cells. (**G**) Representative flow cytometry plots of CD4$^+$ and CD8$^+$ T cells among live, singlet, CD45$^+$, F4/80$^-$, TCRβ^+ cells. (**H-I**) Frequency and number of CD4$^+$ or CD8$^+$ T cells. Data are representative of 2 independent experiments. *P* values were determined by one-way ANOVA with Tukey's multiple comparison test. *, $P < 0.05$; **, $P < 0.01$; ***, $P < 0.001$; ****, $P < 0.0001$.
